# Relational study between sleep quality, daytime sleepiness state, anxiety, stress, depression in possible bruxist and non-bruxist: cross-sectional study

**DOI:** 10.1007/s10266-025-01117-1

**Published:** 2025-05-07

**Authors:** Ana González González, Ana María Martín Casado, Cristina Gómez-Polo

**Affiliations:** 1 Present Address: Department of Dentistry, School of Medicine, University of Salamanca, Campus Miguel de Unamuno S/N, 37007 Salamanca, Spain; 2 Department of Statistics, School of Medicine, University of Salamanca, Salamanca, Spain

**Keywords:** Bruxism, Sleep quality, Anxiety, Depression, Stress, Self-reports

## Abstract

This study aims to analyze whether individuals who self-report as possible bruxism differ from non-bruxism in terms of sleep quality, daytime sleepiness, and emotional disorders (depression, anxiety, and stress). Additionally, it examines the relationships between these emotional disorders, sleep quality, and daytime sleepiness in both groups. An online questionnaire was administered to 400 Spanish participants without cognitive impairments. The questionnaire included self-report measures of sleep bruxism, the Pittsburgh Sleep Quality Index, the Epworth Sleepiness Scale, and the Depression, Anxiety, and Stress Scale-21. Statistical analyses, including the Chi-square test and the *T*-test, were conducted to assess associations. A total of 21.8% of participants self-reported as possible bruxism, with a higher prevalence among women and individuals under 50 years of age. No significant differences were found between bruxism and non-bruxism regarding the percentage of participants with poor sleep quality or excessive daytime sleepiness (*p* > .05). However, significant differences were observed in levels of depression, anxiety, and stress, which were higher among possible bruxism. Possible sleep bruxism is not associated with excessive daytime sleepiness but is linked to poorer sleep quality and greater severity of depression, anxiety, and stress. Stronger associations between sleep quality and both depression and anxiety were observed in the bruxism group compared to non-bruxism, suggesting that psychological disorders have a more consistent impact on sleep quality in bruxism. Women, middle-aged individuals, and those with a university education reported higher rates of bruxism symptoms. Bruxism is associated with poorer sleep quality and psychological disorders such as depression, anxiety, and stress, which negatively impact quality of life. Understanding these associations, their prevalence, and the psychological profile of bruxism can help in designing more effective intervention programs to mitigate these adverse effects.

## Introduction

Sleep is a vital physiological process that occupies approximately 20–40% of a person’s daily schedule. When sleep duration is reduced, it negatively impacts cognitive, emotional, social, and economic functioning [1–3]. However, others argue that sleep quality declines due to increased fragmentation and lighter sleep, which can lead to excessive daytime sleepiness (EDS) [4, 5]. Research on sleep disorders has increased in recent years due to growing interest in their clinical effects [2, 6]. Despite this, there is a global trend of reduced sleep duration, which leads to decreased performance, concentration deficits, increased irritability, and fatigue [7, 8]. Sleep disorders can be influenced by external (social and environmental) factors [9], internal factors such as psychological disorders [10, 11] and lifestyle habits including alcohol consumption, tobacco use, caffeine intake, and electronic device use [10, 11]. Poor sleep is linked to various health conditions, including cardiovascular diseases, type 2 diabetes, respiratory disorders such as sleep apnea, obesity, and parafunctional behaviors like sleep bruxism (SB) [12–14]. SB is defined as “a repetitive activity of the masticatory muscles characterized by clenching or grinding of the teeth and/or by tension or thrusting of the jaw” [15, 16]. SB is considered parasomnia when it is associated with micro-awakenings; otherwise, it is classified as a parafunctional habit [15]. In addition to micro-awakenings, SB can cause chronic pain during the day [17], which in turn may contribute to cognitive issues, insomnia, fatigue, and emotional disorders such as anxiety, depression, and stress [17]. It remains unclear whether psychological disorders develop as a consequence of chronic pain or if they play a role in the onset and persistence of chronic pain [18]. The etiology of SB is multifactorial and primarily associated with complex multisystemic physiological processes, including central dysregulation of motor and neuro-masticatory systems, which influence mandibular muscle activity [19]. In the last decade, research has increasingly focused on the association between SB and concomitant pathologies. The increase in mandibular muscle activity appears to be a reaction to microarousals during sleep, triggered by elevated heart rate and increased muscle tone lasting from 3 to 10 s [14, 20]. The clinical manifestations of SB include damage to muscles, ligaments, joints, and teeth [21]. When SB disrupts sleep, it is often associated with long-term emotional disorders such as anxiety, depression, and stress [22, 23].

Emotional disorders are characterized by increased cortisol levels due to sleep deprivation, yet they also contribute to difficulty in achieving restful sleep. This creates a cycle where it is unclear whether sleep disturbances are the cause or the effect of emotional disorders [24]. These disorders are increasingly prevalent in the adult population of developed countries due to the fast-paced nature of modern life, which includes factors such as overwork, work-life balance challenges, and economic pressures. Psychological distress, in turn, reduces quality of life and manifests in physical symptoms [25, 26]. Stress and anxiety appear to play a role in the pathophysiology of SB [27]. For diagnosis and screening purposes, self-reports through standardized questionnaires for SB, sleep quality, and stress, anxiety, and depression are among the most commonly used methods. While these provide subjective information, they remain widely used due to their low cost and high accessibility. However, self-reports do not always correlate with polysomnography (PSG), the gold standard for diagnosing SB [16, 28]. While PSG is ideal for assessing sleep quality, its high cost and long waiting times make it neither simple nor accessible for many individuals [29]. As a result, the Pittsburgh Sleep Quality Index (PSQI) [30] and the Epworth Sleepiness Scale [31], which quantifies excessive daytime sleepiness (EDS), are more frequently used. Additionally, the Lobbezoo et al. questionnaire [16], is commonly employed to categorize participants as “possible bruxism.”

The relationship between BS, sleep cycle disturbances, and psychological disorders—such as anxiety, depression, and stress—requires further investigation, as the strength of their association remains unclear. Research is needed to determine whether these conditions act as predisposing or perpetuating factors—or potentially both, along with their prevalence across different sociodemographic groups, would allow for more efficient study designs. To address this issue, it is essential to develop more effective diagnostic, prevention, and intervention programs within primary care. Establishing such programs could help minimize prevalence rates and allow for early monitoring of patients, even before the onset of symptoms. Currently, no comprehensive studies have been conducted on the general Spanish population to serve as a reference for prevention, diagnosis, and early treatment protocols for these conditions. Additionally, dentists should be encouraged to acquire the necessary knowledge to assess and identify potential links between BS and other coexisting physical and mental health conditions. Through consistent follow-up, dentists can ensure that patient education extends beyond oral health, providing a more comprehensive understanding of the frequent associations between “possible bruxism” and broader systemic conditions.

The objectives of the present study are as follows: (1) To categorize the study participants as possible bruxism or non-bruxism according to the Lobbezoo et al. test [16] and to compare prevalence rates based on age, gender, educational level and smoking habits, (2) to analyze whether possible bruxism and non-bruxism differ in terms of sleep quality, EDS, and emotional disorders, specifically depression, anxiety, and stress, and (3) to examine and compare the relationship between sleep quality, EDS and the presence of anxiety, stress, and depression in both groups: possible bruxism and non-bruxism. These objectives are accompanied by the following null hypotheses: (1) the prevalence of possible bruxism does not depend on gender, age, educational level, or smoking habits, (2) possible bruxism and non-bruxism do not differ in (a) sleep quality, (b) EDS, (c) any of the emotional disorders of the DASS-21, and (3) The emotional disorders of the DASS-21 are not related to either sleep quality or the level of daytime sleepiness in either group: possible bruxism or non-bruxism.

## Materials and methods

An online questionnaire was created using Google Forms. Respondents were briefly informed about the study’s objectives, and the research approval registration number from the institutional Bioethics Committee (CBE-U.S.A.L Nº1081) was provided. Instructions on how to complete the questionnaire were given according to the guidelines set by the authors of the validated scales. Participants were required to submit a fully completed questionnaire, as unanswered items were automatically flagged for response. The surveys were entirely anonymous. Data collection occurred over a three-month period in 2024. Participants were recruited through a non-probabilistic “snowball” sampling method. Initial contact was made via email and WhatsApp through teaching staff, students, and administrative personnel of the University Dental Clinic who met the inclusion criteria. They were encouraged to share the survey with friends, family, and colleagues, who, in turn, invited additional participants. The survey introduction specified the inclusion criteria: Spanish-speaking adults over 18 years of age with full cognitive abilities and a willingness to participate in the study.

The questionnaire gathered the necessary data for descriptive statistics and incorporated several validated scales. The structure of the questionnaire was as follows:

1. *Sociodemographic data*: Gender, age, educational level, usual living environment, province of residence, and smoking habits.
2. *SB Self-Perception Questionnaire:* The Lobbezoo et al. test [16] test consists of 5 dichotomous (yes/no) items: 3 regarding the occurrence of sleep bruxism (SB) and 2 about the presence of daytime bruxism (DB). A participant is classified as a “possible” bruxism if they respond positively to 3 or more items. This test has demonstrated adequate internal consistency, with Cronbach’s alpha values exceeding the threshold of 0.70, indicating strong inter-item correlation and reliable measurement of bruxism experience. [32] The validation of the Lobbezoo bruxism self-perception test into Spanish is crucial to ensuring its reliability and applicability in both research and clinical settings in Spanish-speaking countries. [33].
3. *Pittsburgh Sleep Quality Index* [30]*:* This scale includes 19 items assessing 7 components of sleep: (1) sleep quality, (2) sleep latency, (3) sleep duration, (4) sleep efficiency, (5) sleep disturbances, (6) use of sleeping pills, and (7) daytime dysfunction. The total score ranges from 0 to 21, with higher scores indicating greater sleep difficulties. Response formats vary, including time intervals (minutes, frequency per week), quality ratings (from “very good” to “very bad”), and severity ratings (from “none” to “serious problem”). A cut-off score of 5 distinguishes good sleepers (≤ 5) from poor sleepers (> 5), with a sensitivity of 89.6% and a specificity of 86.5%. [30]
4. *Epworth Sleepiness Scale (ESS)* [31]: This questionnaire measures the likelihood of falling asleep in various everyday situations. It comprises eight items rated on a Likert-type scale: No chance of falling asleep (0 points) / Slight chance of falling asleep (1 point)/Moderate chance of falling asleep (2 points)/High chance of falling asleep (3 points). Total scores categorize participants as follows: 0–10 points: normal daytime sleepiness, 11–24 points: excessive daytime sleepiness [34].
5. *Depression, Anxiety and Stress Scale (DASS-21)* [35]: This scale consists of 21 items, with 7 items per subscale assessing depression, anxiety, and stress. Participants rate their experiences over the past week on a four-point Likert scale: 0 (Did not apply to me at all), 1 (Applied to me to some degree), 2 (Applied to me to a considerable degree), and 3 (Applied to me very much or most of the time). All the items on each scale are added together to obtain a total score, which ranges from 0 to 21. Subscale scores are summed to yield total scores ranging from 0 to 21. Higher scores indicate greater symptom severity. The classification criteria are as follows: Depression: 5–6 mild, 7–10 moderate, 11–13 severe, 14 or more extremely severe; Anxiety: 4 mild, 5–7 moderate, 8–9 severe, 10 or more extremely severe; stress: 8–9 mild, 10–12 moderate, 13–16 severe, 17 or more extremely severe.

In this study, seasonal disorders were excluded. This decision was intended to ensure that the results would have greater clinical applicability and relevance for identifying clear associations with bruxism. Furthermore, the questionnaires used, such as the DASS-21, are validated to measure persistent emotional disorders, but they do not include specific items related to seasonal patterns, such as seasonal affective disorder. Integrating additional measures of this type would have required different scales and could have increased the complexity of the analysis. The lower relevance of seasonality in the context of bruxism should also be considered. Seasonal cognitive disorders tend to be more related to factors such as sunlight exposure or seasonal changes, while bruxism has a multifactorial etiology more associated with neuromuscular, emotional, and lifestyle processes. Therefore, it may have been less relevant to include these disorders.

### Statistical analysis

The normality of the variables was assessed using the Kolmogorov–Smirnov–Lilliefors test in combination with graphical methods, including frequency histogram and QQ plots. The association between categorical variables was analyzed using the Chi-square test and the Fisher–Freeman–Halton exact [36]. Comparisons of scores between possible bruxism and non-bruxism were conducted using the t-test for independent samples, when the data were approximately normally distributed. When normality assumption was not met, the Mann–Whitney U test was applied. The association between two quantitative variables was examined using Spearman’s rank correlation coefficient [37]. Correlation strength (in absolute value) was interpreted as follows: 0–0.3, negligible correlation; 0.3–0.5, weak correlation; 0.5–0.7, moderate correlation; 0.7–0.9, strong correlation; and greater than 0.9, very strong correlation [38]. All statistical analyses were performed using SPSS v.28 software. The significance level was set at 0.05.

## Results

### Sample description

The sample consisted of 400 voluntary participants who completed the survey online. Of these, 264 were women (66.0%) and 136 were men (34.0%). Participants’ ages ranged from 19 to 84 years, with a mean age of 41.4 years and a standard deviation of 13.6 years. By age group, 80 participants (20.0%) were between 18 and 29 years old, 202 (50.5%) were between 30 and 49 years old, 108 (27.0%) were between 50 and 69 years old, and 10 (2.5%) were 70 years old or older. Regarding educational level, 17 participants (4.3%) had completed primary education, 103 (25.8%) had completed secondary education, and 280 (70.0%) had completed university education. Additionally, 70 participants (17.5%) were smokers, while 330 (82.5%) were non-smokers.

Table 1 presents the frequencies and percentages of the different categories in which the participants were classified based on their responses to the questionnaires administered to the total sample.

**Table 1:** Frequency and percentage of the categories of qualitative variables (total sample, n = 400)

Variable	n	%
*Bruxism (Lobbezoo)*		
Possible bruxism	87	21.8
Non-bruxism	313	78.3
*Sleep quality (categorized)*		
Good	194	49.1
Poor	201	50.9
*Daytime sleepiness (categorized)*		
Normal	282	70.5
Excessive	118	29.5
*Depression (categorized)*		
No depression	280	70.0
Mild depression	41	10.3
Moderate depression	46	11.5
Severe depression	18	4.5
Extremely severe depression	15	3.8
*Anxiety (categorized)*		
No anxiety	277	69.3
Mild anxiety	32	8.0
Moderate anxiety	51	12.8
Severe anxiety	20	5.0
Extremely severe anxiety	20	5.0
*Stress (categorized)*		
No stress	284	71.0
Mild stress	40	10.0
Moderate stress	43	10.8
Severe stress	27	6.8
Extremely severe stress	6	1.5

As shown in Table 1, 21.8% of participants identified themselves as possible bruxism. Additionally, a notably high percentage (50.9%) reported poor sleep quality. While lower, substantial proportions also experienced excessive daytime sleepiness (EDS) (29.5%), some level of depression (30.0%), anxiety (30.7%), and stress (29.0%).

### Relationship between possible bruxism and gender, age, educational level and smoking habits

Table 2 presents the characterization of the two groups -possible bruxism and non-bruxism- based on gender, age, educational level, and smoking habits (smoker/non-smoker).

**Table 2:** Characterization of possible bruxism and non-bruxism according to gender, age group, educational level, and smoking habits, and P value of the independence tests

	Possible bruxism	Non-bruxism	P value
*Gender*			0.014
Men	20 (14.7%)	116 (85.3%)	
Women	67 (25.4%)	197 (74.6%)	
*Age group*			< 0.001
18–29 years	21 (26.2%)	59 (73.8%)	
30–49 years	56 (27.7%)	146 (72.3%)	
50–69 years	10 (9.3%)	98 (90.7%)	
70 + years	0 (0.0%)	10 (100.0%)	
*Educational level*			0.017
Primary	0 (0.0%)	17 (100.0%)	
Secondary	18 (17.5%)	85 (82.5%)	
University	69 (24.6%)	211 (75.4%)	
*Smoker*			0.089
Yes	20 (28.6%)	50 (71.4%)	
No	67 (20.3%)	263 (79.7%)	

Table 2 indicates that 14.7% of men reported possible bruxism, whereas the percentage among women was notably higher at 25.4%. This difference is statistically significant. In terms of age, younger participants (18–49 years) exhibited a higher prevalence of possible bruxism compared to older participants, with the association between Lobbezoo test results and age also reaching statistical significance. Regarding educational level, the proportion of possible bruxism increased with higher levels of education. Notably, no participants with only primary education were classified as possible bruxism, whereas among university-educated participants, 1 in 4 reported possible bruxism. Finally, while a greater percentage of smokers (28.6%) reported possible bruxism compared to non-smokers (20.3%), this difference was not statistically significant. Additionally, Table 2 reveals that among possible bruxism, 77.0% were women (67 out of 87), and 88.5% were under the age of 50 (77 out of 87).

### Differences in sleep quality, excessive daytime sleepiness, and emotional disorders (DASS-21) between possible bruxism and non-bruxism

Table 3 presents comparisons of sleep quality, excessive daytime sleepiness (EDS), and emotional disorders between possible bruxism and non-bruxism.

**Table 3:** Association between possible bruxism (yes/no) and the categorical variables: sleep quality, daytime sleepiness, depression, anxiety and stress

	Possible bruxism	Non-bruxism	P value
	(n = 87)	(n = 313)	
*Sleep quality*			0.128
Good	36 (41.9%)	158 (51.1%)	
Poor	50 (58.1%)	151 (48.9%)	
*Daytime sleepiness-ESD*			0.156
Normal	56 (64.4%)	226 (72.2%)	
Excessive	31 (35.6%)	135 (27.8%)	
*Depression*			< 0.001
No depression	48 (55.2%)	232 (74.1%)	
Mild depression	8 (9.2%)	33 (10.5%)	
Moderate depression	16 (18.4%)	30 (9.6%)	
Severe depression	5 (5.7%)	13 (4.2%)	
Extremely severe depression	10 (11.5%)	5 (1.6%)	
*Anxiety*			0.002
No anxiety	46 (52.9%)	231 (73.8%)	
Mild anxiety	8 (9.2%)	24 (7.7%)	
Moderate anxiety	18 (20.7%)	33 (10.5%)	
Severe anxiety	6 (6.9%)	14 (4.5%)	
Extremely severe anxiety	9 (10.3%)	11 (3.5%)	
*Stress*			< 0.001
No stress	47 (54.0%)	237 (75.7%)	
Mild stress	9 (10.3%)	31 (9.9%)	
Moderate stress	16 (18.4%)	27 (8.6%)	
Severe stress	12 (13.8%)	15 (4.8%)	
Extremely severe stress	3 (3.4%)	3 (1.0%)	

Among possible bruxism, 58.1% reported poor sleep quality, compared to 48.9% among non-bruxism. However, this difference was not statistically significant (P > 0.05). Similarly, the proportion of possible bruxism with excessive daytime sleepiness (35.6%) was higher than that of non-bruxism (27.8%), but this difference also lacked statistical significance (P > 0.05). In contrast, there was a statistically significant association between possible bruxism (yes/no) and depression, possible bruxism (yes/no) and anxiety, and possible bruxism (yes/no) and stress. Across all three emotional disorders, the percentage of participants with moderate and severe degrees of these disorders was higher in the group of possible bruxism than in the group of non-bruxism (Table 3).

Table 4 presents the results obtained when, instead of categorizing sleep quality and daytime sleepiness, the total scores from the Pittsburgh and Epworth tests were used to compare these variables between possible bruxism and non-bruxism.

**Table 4:** Comparison of sleep quality and daytime sleepiness scores between possible bruxism and non-bruxism (independent samples t-test)

	Possible bruxism (n = 87)	Non-bruxism (n = 313)	P value
	Mean (SD)	Mean (SD)	
Sleep quality	6.9 (3.3)	6.1 (3.3)	0.045
Daytime sleepiness	9.0 (5.3)	8.2 (4.5)	0.244

Possible bruxism had statistically significantly poorer sleep quality than non-bruxism, but no significant differences were observed in EDS. When comparing the scores of the 7 components of the PSQI [30] using the Mann–Whitney test, statistically significant differences (P = 0.024) were found only in the use of hypnotic medication (component 6), with higher scores among possible bruxism.

### Analysis of the relationship between psychological disorders on the DASS-21 and sleep quality and EDS in each group of participants (possible bruxism/non-bruxism)

Table 5 presents the Spearman correlation coefficients between the scores on the 3 subscales of the DASS-21 (depression, anxiety and stress) [35] and the scores on the Pittsburgh Sleep Quality Index (sleep quality) [30] and the Epworth Sleepiness Scale (daytime sleepiness) [31] for both possible bruxism and non-bruxism.

**Table 5:** Association between sleep quality and daytime sleepiness with depression, anxiety and stress in possible bruxism and in non-bruxism

	Possible bruxism			Non-bruxism		
	Depression	Anxiety	Stress	Depression	Anxiety	Stress
*Sleep quality*	0.411**	0.408**	0.407**	0.313**	0.348**	0.439**
Comp. 1: Subjective quality	0.271*	0.382**	0.330**	0.226**	0.280**	0.406**
Comp. 2: Sleep latency	0.314**	0.245*	0.318**	0.181**	0.280**	0.237**
Comp. 3: Sleep duration	− 0.087	− 0.065	− 0.034	0.098	0.034	0.195**
Comp. 4: Sleep efficiency	0.045	0.076	0.047	0.124*	0.141*	0.240**
Comp. 5: Sleep disturbances	0.123	0.235*	0.137	0.230**	0.329**	0.260**
Comp. 6: Use of hypnotic medication	0.321**	0.255*	0.279**	0.222**	0.273**	0.258**
Comp. 7: Daytime dysfunction	0.523**	0.454**	0.495**	0.348**	0.239**	0.375**
*Daytime sleepiness*	0.013	0.096	0.031	0.131*	0.104	0.073

*0.01 < P < 0.05; **P < 0.01

Sleep quality is positively correlated with all 3 emotional disorders assessed by the DASS-21 [35] in both possible bruxism and in non-bruxism. This indicates that higher symptomatology in any of the 3 disorders is associated with poorer sleep quality in both groups. The associations between depression, anxiety, and stress with overall sleep quality scores are weak (between 0.3 and 0.5) [38], yet statistically significant. Among possible bruxism, emotional symptoms are positively correlated with subjective sleep quality, sleep latency, use of hypnotic medication and daytime dysfunction. In contrast, among non-bruxism, emotional symptoms are positively correlated with all components of sleep quality, except for sleep duration, which is only positively correlated with stress scores.

Daytime sleepiness (DSS) does not correlate with any of the 3 emotional disorders in possible bruxism and is only positively correlated with depression in non-bruxism. Although statistically significant, the association between daytime sleepiness and depression is negligible (less than 0.3) [38]. Table 5 also indicates that the correlations between depression and sleep quality, as well as between anxiety and sleep quality, are weaker in non-bruxism than in possible bruxism. However, the correlation between stress and sleep quality is slightly stronger in non-bruxism compared to possible bruxism.

## Discussion

Current research does not establish a consistent prevalence rate for possible bruxism, with estimates ranging from as low as 5% [39] to as high as 37.5% [40]. This wide range makes it difficult to determine the true frequency of bruxism in the general population. The variability in reported prevalence is largely attributed to population biases in study designs [41, 42], the challenges of accurately diagnosing bruxism using methods such as electromyography [43] or polysomnography (PSG) for sleep bruxism [44], and the predominance of cross-sectional studies [45, 46]. Additionally, many studies rely on self-reported data, which can lead to inaccuracies [47].

There is also no consensus on the most appropriate self-perception test to assess bruxism. The 2018 International Consensus endorsed the Lobbezoo et al. test [16], but the test developed by Fierro et al. [33], is considered more comprehensive, as it incorporates questions from multiple existing assessments. Self-reported methods often lead to an overestimation of daytime bruxism while underdiagnosing sleep bruxism, as individuals are typically only aware of their daytime habits [48]. Unless sleep bruxism is particularly severe -resulting in audible grinding detected by a bed partner- the individual may experience the consequences of the condition but remain unaware of its occurrence [49]. Further complicating the issue, research does not always differentiate between types of bruxism. Some studies group all forms together (Sutin et al., 2010), while others focus exclusively on awake bruxism [50] or sleep bruxism [51].

In this study, emotional disorders such as anxiety, depression, and stress were present in approximately 30% of the sample: depression (30.0%), anxiety (30.7%), and stress (29.0%). The rise in mental health problems can be attributed to multiple factors, including the recent global COVID-19 pandemic [52], which led to social isolation, work stress, and economic concerns [53]. Additionally, social-technological changes [54], particularly the increasing demand for constant connectivity to electronic devices, have played a role. Other contributing factors include personal relationship difficulties, the loss of loved ones, and unexpected life events [55].

### Relationship between possible bruxism, gender, age, educational level and smoking habits

Regarding the presence of bruxism by gender, the data from this study indicate that women are more likely to be aware of and self-report the condition. A total of 67 women (77.0%) compared to 20 men (23.0%) self-reported as possible bruxism. However, the literature presents conflicting findings. Some studies with sample sizes similar to ours have found no significant gender differences [56], whereas others, particularly those with larger sample sizes (over 4000 respondents), report a higher prevalence of bruxism among women [57].

Regarding age, the findings of this study suggest that bruxism is less prevalent from the age of 50 onwards, whereas younger and middle-aged adults are more likely to self-report possible bruxism. Some studies support these findings [58], yet, once again, the literature presents inconsistent data. These discrepancies may be attributed to differences in study design, sample selection biases [59, 60], or variations in the types of bruxism analyzed [61]. The results suggest that a typical profile for a possible bruxism may be a woman under 50 years of age, which highlights the importance of targeted screening in this population group to assess potential risk. The higher prevalence of bruxism in women compared to men can be explained by several factors: (1) Stress and anxiety: Women tend to report higher levels of stress and anxiety, which are key risk factors for bruxism. Studies have shown that people with anxiety are up to three times more likely to develop bruxism [62]. (2) Hormonal factors: Hormonal changes, such as those related to the menstrual cycle, pregnancy, or menopause, can influence the onset of bruxism. These changes can affect sleep quality and increase muscle tension [63]. (3) Greater self-awareness: Women are often more aware of their health habits and more likely to seek medical help, which could lead to a higher rate of self-reported bruxism [64]. (4) Differences in pain perception: Some studies suggest that women may be more sensitive to muscle and joint pain associated with bruxism, which could make them more likely to identify and report the problem [65].

With respect to educational level, individuals with higher levels of education appear to be more aware of their bruxism symptoms. However, in this study, the sample is imbalanced, with very few participants from lower educational backgrounds, making it difficult to draw firm conclusions. Further research is needed to confirm this potential relationship.

Finally, this study examined possible differences in bruxism prevalence between smokers and non-smokers. No significant differences were found, suggesting that smoking does not appear to be a determining factor in the prevalence of bruxism [66].

### Differences in sleep quality, excessive daytime sleepiness, and emotional disorders of the DASS-21 between possible bruxism and non-bruxism

One factor affecting sleep quality in modern lifestyles is the increasing use of new technologies. Screen time is rising, which disrupts circadian rhythms and negatively impacts both sleep quantity and quality [67]. Despite this widely acknowledged issue, there is still no consensus within the scientific community, as research methods vary considerably and findings remain inconclusive [68].

The Pittsburgh Sleep Quality Index (PSQI) revealed significant differences when comparing the overall sleep quality scores of possible bruxism and non-bruxism. This finding aligns with previous studies [69, 70]. However, when categorizing participants as having either good or poor sleep quality (with a PSQI score > 5 indicating poor sleep quality), no statistically significant differences were found between the two groups. To address this, a component-by-component analysis was conducted to determine which aspects of sleep disturbances were most affected in the group of possible bruxism. The results indicate that possible bruxism generally report worse sleep quality and are more likely to require medication to fall asleep [71]. The PSQI cutoff score showed no significant differences between the possible bruxist and non bruxist groups due to several methodological and statistical factors: (1) Loss of information when categorizing: the PSQI uses a cutoff score of 5 to classify participants as “good” or “poor” sleepers. This simplification may obscure more subtle differences in sleep quality between groups, especially if scores are close to the cutoff and if the categories do not adequately reflect the underlying data. (2) Variability reduction: although possible bruxers reported slightly worse sleep quality on average, variability within each group could have reduced the ability to detect significant differences in binary categorization when total PSQI scores were analyzed, possible bruxers showed significantly worse sleep quality than non-bruxers, suggesting that differences may be more evident in a continuous analysis than in a categorical one. Furthermore, bruxers reported greater use of sleep medication, which could be a proxy for more severe sleep problems. Excessive daytime sleepiness (EDS) did not show significant differences between respondents who self-reported as possible bruxism and those who did not. The relationship between possible bruxism and psychological disorders was also examined. A significant correlation was found only between depression and non-bruxism, while anxiety and stress did not show strong associations. Sleep bruxism (SB) may contribute to reduced sleep quality, and the resulting muscle pain and discomfort could exacerbate feelings of discouragement and fatigue [72]. Participants with moderate levels of emotional disorders (specifically anxiety, depression and stress) showed a significantly higher prevalence of self-reported bruxism compared to those without these psychological conditions. The relationship between psychological disorders and BS also extends to pain severity. Psychological disorders can amplify pain perception, and chronic pain can exacerbate psychological symptoms. Chronic stress can constantly activate the hypothalamic–pituitary–adrenal axis, leading to elevated cortisol levels. This not only affects emotional regulation but can also sensitize pain pathways, increasing their intensity and duration. Patients with higher levels of anxiety or depression tend to report more severe pain in temporomandibular disorders [73]. Poor sleep quality can trigger a detrimental cycle that affects both mental and physical health, increasing the risk of long-term painful complications. During sleep, the brain processes emotions and regulates stress. A lack of restful sleep can intensify anxiety, depression, and stress, which in turn affects pain perception and can amplify it. Sleep deprivation is also linked to elevated levels of inflammation in the body and can disrupt the neurological systems that regulate pain perception, making people more sensitive to painful stimuli. Painful conditions, such as bruxism or chronic pain, can further disrupt sleep, creating a vicious cycle where pain and lack of sleep reinforce each other, affecting quality of life and hindering recovery [74].

### Relationship between psychological disorders of DASS-21 and sleep quality and EDS in each group of participants (possible bruxism/non-bruxism)

Psychological factors were measured using the DASS-21 scale, which evaluates anxiety, depression, and stress. These factors were analyzed separately to assess each pathology independently. The results indicate that participants who self-reported as possible bruxism had higher scores on the DASS-21 scale, indicating greater levels of depression, anxiety, and stress. Furthermore, these disorders showed a statistically significant correlation with poor sleep quality [75], although the associations were weak. Conducting research on psychosocial factors requires complex and often controversial methodologies, which is why questionnaires are commonly used. However, findings from such studies are frequently contradictory [76]. In this study, stronger associations between sleep quality and depression, as well as between sleep quality and anxiety, were observed in the group of possible bruxism compared to the group of non-bruxism. This suggests that these psychological disorders have a more consistent impact on sleep quality in individuals who self-report bruxism. The findings highlight that bruxism is not only a physical condition but also significantly affects mental health, emphasizing the need to address both the physical and emotional aspects in bruxism treatment.

Psychological factors play a crucial role in numerous pathologies, as emotions can manifest in physical symptoms [61]. The connection between mind and body remains an area that has not yet been fully explored. More research of this kind, which considers both physical signs and emotional symptoms, could help develop new explanations for the etiologies and progression of various disorders.

Efforts should be made to improve coping mechanisms so that individuals are less susceptible to anxiety and stress [77]. Age significantly influences the prevalence of psychological disorders, as different life stages present distinct challenges [78]. Psychological inequalities impact an individual’s ability to manage stressful situations, their physical and mental repercussions, and their overall vulnerability [79]. A potential physical manifestation of these psychological challenges is the development of bruxism. However, studies on bruxism prevalence across different age groups yield conflicting results—some suggest it is more common in younger individuals [41], while others claim that its prevalence increases with age [80].

Although mental health is increasingly prioritized in disorder prevention, standardized diagnostic criteria remain insufficient. Researchers struggle to obtain reliable data, as diagnostic methods often rely on subjective self-reporting tools such as questionnaires [81–83]. More precise diagnostic methods for bruxism and sleep disturbances, such as polysomnography or electromyography, are costly and have long waiting lists. As a result, many patients remain underdiagnosed, while those who receive a diagnosis often face significant delays in treatment. Given the well-established link between psychological factors and pain-related conditions, preliminary screening could incorporate surveys or simple tests, such as saliva cortisol analysis, to enable earlier intervention. A multidisciplinary approach could help mitigate these conditions more effectively by addressing both physical and psychological factors.

Emotional disorders, such as anxiety, depression, and stress, tend to affect overall sleep quality more than daytime sleepiness due to how they influence the brain's nighttime processes. During sleep, the brain regulates emotions and processes emotional information, which can be disrupted by these disorders. This leads to decreased sleep quality, such as interruptions or difficulty falling asleep, but does not necessarily cause daytime sleepiness, which is more related to factors such as total sleep duration or specific conditions such as sleep apnea [84]. For age and gender groups, differences in sleep quality may be due to how each individual experiences and manages stress or anxiety, which directly affects nighttime sleep patterns. However, daytime sleepiness may be more influenced by other physiological factors or daily habits, such as the use of electronic devices or caffeine consumption, which are not always directly linked to emotional disorders [85].

### Strengths and limitations of the study

This study provides several interesting and novel findings compared to previous research on bruxism and its relationship with sleep quality and emotional disorders: (1) Association between bruxism and emotional disorders: Although previous research has already pointed to a connection between bruxism and disorders such as anxiety and depression, this study highlights that these associations are stronger in people with bruxism compared to those without the condition. This suggests that emotional disorders have a more consistent impact on the sleep quality of bruxers, (2) Differences in sleep quality: The study confirms that suspected bruxers have significantly worse sleep quality than non-bruxers. However, no significant differences were found in excessive daytime sleepiness between the two groups, a finding that contrasts with some previous studies, (3) Sociodemographic profile of bruxism: This study identifies that women and people under 50 years of age have a higher prevalence of bruxism, reinforcing the need for targeted screening approaches in these groups, (4) Use of hypnotic medication: A specific finding is that bruxers report greater use of sleep medications, which could indicate greater difficulty falling asleep in this group and (5) Relationship between sleep quality and emotional disorders: Sleep quality was found to be correlated with levels of depression, anxiety, and stress in both groups, but these correlations were stronger in bruxers. This underscores the importance of addressing both physical and emotional aspects in bruxism treatment. These results not only expand existing knowledge but also highlight the need for a multidisciplinary approach to the diagnosis and treatment of bruxism, considering both physical and psychological factors, especially among women, young adults, and people with higher educational levels. This could guide more integrated prevention and intervention strategies.

However, this study also has limitations such as: (1) This is a cross-sectional study that explores the connections between sleep quality, daytime sleepiness, and emotional disorders (anxiety, stress, and depression) in people who call themselves possible bruxers, compared to those who do not. In cross-sectional studies, it is difficult to determine the chronological order among the variables studied and, therefore, it is not possible to establish cause-effect relationships. Moreover, there may be additional factors (confounding variables), not considered in the study, that influence the relationships between the variables analyzed and alter the perception of temporality; alcohol consumption and the use of certain medications, among others, could be mentioned, (2) The study used a non-probability snowball sampling method, where the initial participants invited others. This approach may have favored greater female representation, given that, according to the literature, women tend to report more frequently conditions such as bruxism and sleep-related emotional problems. Furthermore, women may be more willing to participate in health or psychological studies, as is often indicated in research related to gender and participation [86]. The type of sampling used may result in the sample not being representative of the general population and the findings may not be generalizable and (3) There are several early diagnosis methods to identify signs of sleep deprivation and psychological factors. This study uses the validated PSQI, ESS and DASS-21 questionnaires. Measures based on self-reports have limitations that may influence the accuracy and validity of the data. Nevertheless, questionnaires are an early detection method with many advantages. They are a cost-effective tool for collecting data from large population samples. Compared to other methods such as interviews or observational studies, questionnaires allow information to be obtained in a shorter period of time. Being validated ensures that the data collected is consistent and comparable across different studies. Furthermore, their structure facilitates statistical analysis. Another advantage is that they promote an anonymous environment, which can encourage participants to be more honest, especially on sensitive topics such as mental health or sleep habits. Tests such as actigraphy, a wearable device that records movements during sleep to assess sleep–wake patterns, could also be used. Sleep tracking devices should also be considered: wristbands or mobile apps that monitor basic parameters such as sleep duration and cycles. Although they are less accurate, they are useful for regular monitoring.

## Conclusions

With the limitations of this study, the following conclusions can be drawn:

1. Women and younger individuals (under 50 years of age) report themselves as possible bruxism more frequently than men and older individuals (over 50 years of age). Therefore, the first null hypothesis is rejected. The prevalence of bruxism is influenced by gender and age.
2. Possible bruxism exhibit a poorer sleep quality and higher levels of depression, anxiety, and stress compared to non-bruxism. However, no significant differences were found in daytime sleepiness between the two groups. This suggests that while bruxism is associated with mental health issues and sleep disturbances, it is not necessarily linked to excessive daytime sleepiness. Consequently, the second null hypothesis is rejected. Possible bruxism differ significantly from non-bruxism in terms of sleep quality and levels of depression, anxiety, and stress, but not in daytime sleepiness.
3. A significant relationship exists between sleep quality and emotional disorders (depression, anxiety and stress) in both possible bruxism and non-bruxism. However, this relationship is stronger in the bruxism group. Daytime sleepiness does not show a significant association with emotional disorders in either group. As a result, the third null hypothesis is rejected. Emotional disorders (depression, anxiety and stress) are significantly related to sleep quality but not to daytime sleepiness in both groups.

## Data Availability

The availability of the data is available upon reasoned request.
